# Acetogenins-Rich Fractions of *Annona coriacea* Suppress Human Glioblastoma Viability and Migration by Regulating Necroptosis and MMP-2 Activity In Vitro

**DOI:** 10.3390/molecules28093809

**Published:** 2023-04-29

**Authors:** Lorena R. Sousa, Ana Gabriela S. Oliveira, Antônio Arantes, João Gabriel M. Junqueira, Gerso P. Alexandre, Vanessa G. P. Severino, Rui Manuel Reis, Bonglee Kim, Rosy I. M. A. Ribeiro

**Affiliations:** 1 Experimental Pathology Laboratory, Federal University of São João del Rei (UFSJ), 400, Sebastião Gonçalves Coelho, Chanadour, Divinópolis 35501-296, MG, Brazil; 2 Institute of Chemistry, Federal University of Goiás (UFG), University Campus, Goiânia 74968-755, GO, Brazil; 3 Molecular Oncology Research Center, Barretos Cancer Hospital, Barretos 14784-400, SP, Brazil; 4 Life and Health Sciences Research Institute (ICVS), School of Medicine, University of Minho, 4710-057 Braga, Portugal; 5 ICVS/3B’s—PT Government Associate Laboratory, 4710-057 Braga, Portugal; 6 ICVS/3B’s—PT Government Associate Laboratory, 4805-017 Guimarães, Portugal; 7 College of Medicine, Kyung Hee University, Seoul 02453, Republic of Korea

**Keywords:** acetogenins, GBM, natural product, Annonaceae, mechanism of death, gliomas, matrix metalloproteinase, necroptosis, *Annona coriacea*

## Abstract

Glioblastoma (GBM) is an incurable primary brain tumor with a poor prognosis. Resection, radiation therapy, and temozolomide (TMZ) are insufficient to increase survival, making the treatment limited. Thus, the search for more effective and specific treatments is essential, making plants a promising source for elucidating new anti-glioblastoma compounds. Accordingly, this study investigated the effects of four fractions of hexane and ethyl acetate extract of *Annona coriacea* Mart., enriched with acetogenins, against GBM cell lines. All four fractions were selectively cytotoxic to GBM cells when compared to TMZ. Moreover, *A. coriacea* fractions delayed cell migration; reduced cytoplasmic projections, the metalloproteinase 2 (MMP-2) activity; and induced morphological changes characteristic of necroptosis, possibly correlated with the increase in receptor-interacting protein kinase 1 and 3 (RIP-1 and RIP-3), apoptosis-inducing factor (AIF), and the non-activation of cleaved caspase 8. The present findings reinforce that fractions of *A. coriacea* Mart. should be considered for more studies focusing treatment of GBM.

## 1. Introduction

GBM is one of the most invasive and aggressive primary brain tumors of human adults. Currently, the treatment of patients consists of surgery followed by radiotherapy and chemotherapy with TMZ [1]. Despite gold-standard treatment, the median patient survival rate remains at just 14 months, partially due to the highly invasive and infiltrative nature of this cancer [2]. Studies focusing on the mechanisms of GBM invasion suggest a strong correlation of this process with the activity of matrix metalloproteinases (MMPs), which degrade components of the extracellular matrix (ECM) such as collagen, fibronectin, and proteoglycans facilitating cell migration [2]. MMPs, especially MMP-2 and MMP-9, have been implicated in playing a critical role in glioma invasiveness, recurrence, and poor prognosis [3]. Considering this scenario, studies aimed at screening new antineoplastic compounds for clinical use against GBM are justified.

*Annona coriacea* Mart (Araticum) is a native plant from Brazilian Cerrado, which presents a great variety of secondary metabolites with a broad spectrum of biological activities such as insecticide, antiprotozoal, antiproliferative, anticholinesterase, anti-inflammatory, as well as anticancer [4]. The acetogenins are a class of secondary metabolites, considered Annonaceae family markers [5], which attracted attention due to their antitumoral properties [6]. Acetogenins can act on the mitochondrial electron transport chain blocking complex I and decreasing the production of adenosine triphosphate (ATP), leading tumor cells to death [7]. Despite some promising results [8], not all antitumor mechanisms of acetogenins have been clarified yet, especially against GBM. Therefore, our study aimed to evaluate the antineoplastic potential of A. coriacea fractions enriched with acetogenins against two GBM cell lines in vitro.

## 2. Results

### 2.1. The Cell Viability of GBM Strains Decreased after Being Exposed to A. coriacea Fractions

**Figure 1:**
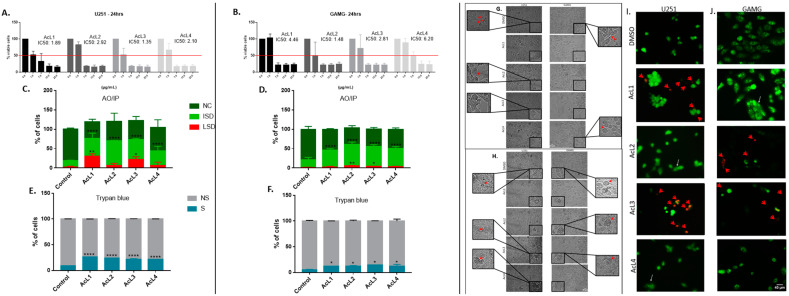
***Annona coriacea* fractions reduced GBM cells viability and induced necroptosis characteristics:** Cell viability by MTT assay of U251 (**A**) and GAMG (**B**). Normal cells (NC) and stages of death (ISD = initial stage of death; LSD = late stage of death) were quantified by AO/PI staining for U251 (**C**) and GAMG (**D**). Trypan blue assay analysis for U251 (**E**) and GAMG (**F**) (NS = not stained; S = stained). Morphological alterations: (**G**) Reduced cytoplasmatic extensions. (**H**) Morphological changes characteristic of cell death. (**I**,**J**) Microphotograph of cell morphology during AO/PI staining; white arrows indicate cells with the characteristic of necroptosis, such as loss of membrane permeability and cell swelling; red arrows indicate cytoplasm leakage. Control: DMSO 1%. Differences between groups were statistically significant with * *p* < 0.05; ** *p* < 0.01; **** *p* < 0.0001. Scale bar = 20 µm.

The toxicity of *A. coriacea* fractions against GBM cells was measured by MTT, AO/PI, and trypan blue assay. The MTT results showed that all the fractions were highly cytotoxic, presenting values of IC_50_ under 7 µg/mL for both cell lines (Figure 1A,B). Furthermore, the AO/PI and trypan blue assay confirm these findings. All four fractions increased the number of cells in the early stages of death for both cell lines and increased the number of those in the late stages of death for the two more cytotoxic fractions (Figure 1C,D). Moreover, all the fractions significantly increased the number of cells stained with trypan blue (Figure 1E,F).

### 2.2. A. coriacea Fractions Increased Morphological Alterations Associated with Necroptosis on GMB Cells Lines

After cell exposure, we observed reductions in lamellipodia, pseudopods, and filopodia in GBM cells (Figure 1G). In addition, we also observed typical morphological features of necroptosis, such as cell swelling, granulations (Figure 1H), and changes in membrane permeability (Figure 1I), that were more remarkable after treatment with the AcL3 fraction for both strains.

### 2.3. A. coriacea Fractions Inhibit Cells Migration of GBM Cell Lines

**Figure 2:**
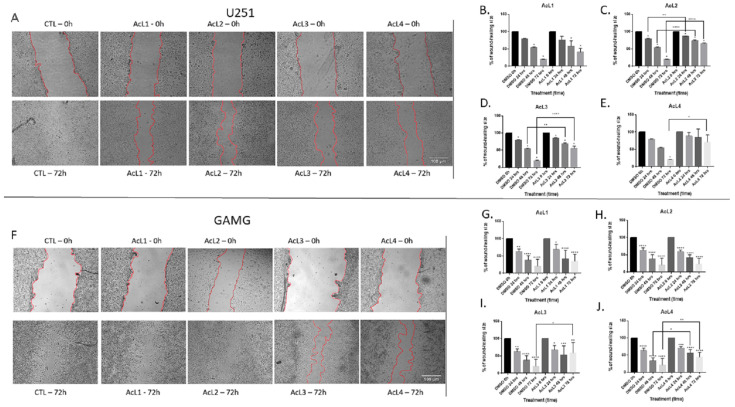
**GBM treatment with *A. coriacea* fractions reduced cell migration:** Representative images from the wound healing assay of U251 (**A**) and GAMG (**F**) cell lines. Analysis of the effect of AcL1, AcL2, AcL3, and AcL4 treatments on the wounds of cell lines U251 (**B**–**E**, respectively) and GAMG (**G**–**J**, respectively). Control: 1% DMSO. Scale bar = 100 µm. Differences between groups were statistically significant with * *p* < 0.05; ** *p* < 0.01; *** *p* < 0.001; **** *p* < 0.0001.

**Figure 3:**
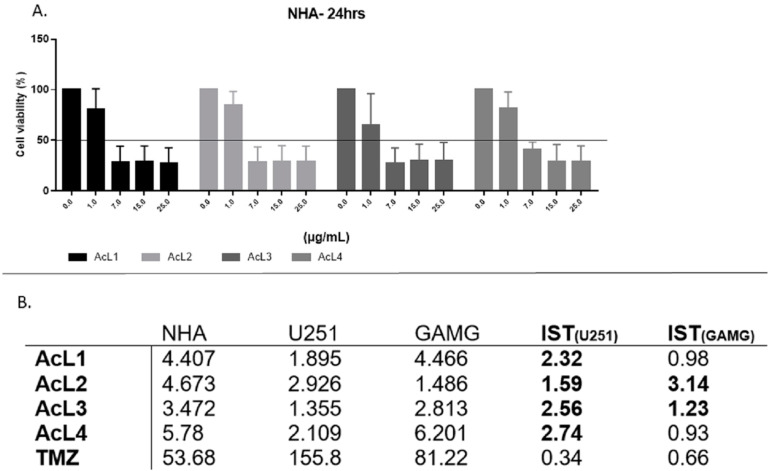
**GBM cells were more affected after treatment with *A. coriacea* fractions than normal astrocytes:** (**A**) Cell viability for NHA after 24 h of treatment with *A. coriacea* fractions. (**B**) Summarized data of IC_50_ values (µg/mL) and corresponding SI for U251 and GAMG. Selectivity index (SI) is the ratio of IC_50_ values of normal cells to those in tumoral cells. SI > 1 is in bold. TMZ data.

All the fractions, except AcL1 for both lineages (2B and 2G) and AcL2 for GAMG (3H), were able to reduce cell migration (Figure 2A–J). For e U251 (Figure 2A), AcL2 showed more promising results, reducing cell migration 24 h after treatment (Figure 3C); followed by AcL3, which significantly reduced cell migration after 48 h of treatment (Figure 2D). However, for GAMG (Figure 2F), AcL4 showed better results, reducing cell migration 48 h after treatment (Figure 2J); followed by AcL3, which significantly reduced cell migration at 72 h after treatment (Figure 2I).

### 2.4. A. coriacea Fractions Is Selective for GBM Cell Lines

Although the *A. coriacea* fractions were cytotoxic to NHA, the IC_50_ values were higher than those found for tumoral cells. Additionally, all fractions were more selective than TMZ for both lineages. For U251, all the SI found were higher than 1, but for GAMG, only AcL2 and AcL3 showed SI > 1 (Figure 3B). AcL3 was the second most selective fraction for both lineages, with SI = 2.56 for U251 and 1.23 for GAMG.

### 2.5. Acetogenins Are Present in All Fractions

**Figure 4:**
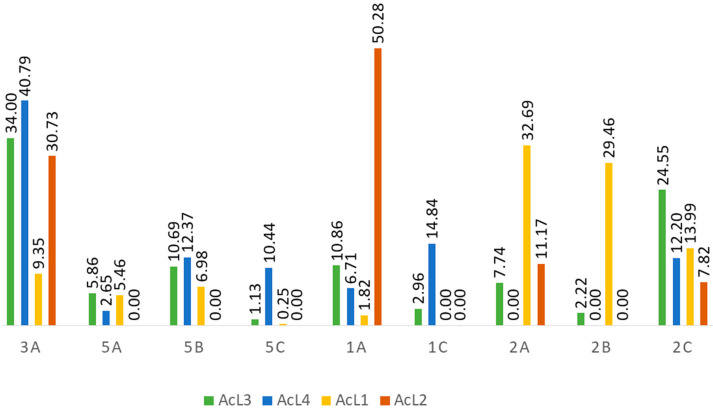
**Percentage area of the compounds identified in the fractions.**

Acetogenins compounds (1a–c, 2a–c, 3a–b isomers) were identified in the four samples (Table 1); 5a–c isomers [9] are present in three samples (AcL1, AcL3, and AcL4). Regarding the relative areas of the compounds identified (Figure 4), the main constituents of the most active fraction (AcL3) are 1a, 2c, 3a, and 5b isomers (80.10%). Although chemical constituents with a lower percentage of the relative area may also have contributed to the antitumor activity. Fraction AcL3 presents greater relative areas for 2c and 3a isomers (Table 2).

### 2.6. A. coriacea Fractions Inhibited Activity but Do Not Alter MMP-2 Expression on GBM Cells

**Figure 5:**
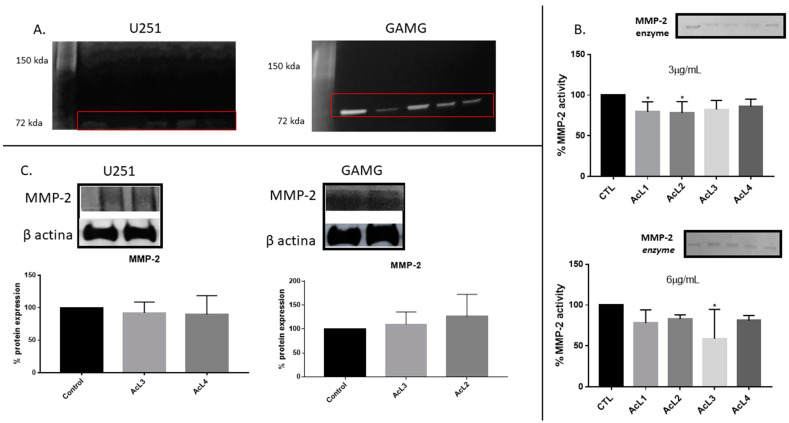
***A. coriacea* treatment inhibited MMP-2 activity, but it did not change its expression: (A) The MMP-2 secreted activity by U251 and GAMG.** (**B**) Action of *A. coriacea* fractions on the MMP-2 enzyme. The fractions AcL1 and AcL2 at the lowest concentration (3 µg/mL) and AcL3 at the highest concentration (6 µg/mL) were significantly able to reduce MMP-2 activity. (**C**) Western blotting analysis did not show significant changes in MMP-2 protein expression by cell lines. Differences between groups were statistically significant with * *p* < 0.05.

GBM cells secreted only MMP-2 (Figure 5A), no band corresponding to MMP-9 activity was observed. Additionally, we investigated the action of the *A. coriacea* fraction on the MMP-2 enzyme (Figure 5B). Only AcL1 and AcL2 in the lower concentration (3 µg/mL) and AcL3 in the upper concentration (6 µg/mL) were capable of reducing the activity of the enzyme significantly. Additionally, the Western blotting analysis showed that none of the fractions altered the expression of MMP-2 (Figure 5C). Therefore, MMP-2 activity was inhibited, as shown on Zymography, but its expression was not altered, as Western blotting analysis showed, after *A. coriacea* fractions treatment.

### 2.7. A. coriacea Fractions Induced Superexpression of RIP-1 on GBM Cell Lines

**Figure 6:**
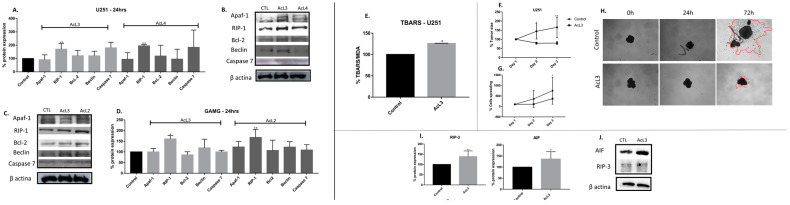
*A. coriacea* fraction induced necroptosis by ROS production and superexpression of RIP-1, RIP-3 and AIF. Expression of proteins involved on cell death pathway for U251 (**A**,**B**) and GAMG (**C**,**D**). Apaf-1 (130 kDa), Bcl-2 (26 kDa), Caspase-7 (35 kDa): apoptosis. RIP-1 (78 kDa): necroptosis. Beclin (60 kDa): autophagy. Results after U251 treatment with AcL3 (**E**) MDA expression; (**F**) Spheroids size; (**G**) Cells spreading analysis; (**H**) Images of tumor size and cells spreading; (**I**) Expression of necroptotic proteins, AIF and RIP-3; (**J**) ꞵ-actin was used as the internal standard. Differences between groups were statistically significant with * *p* < 0.05; ** *p* < 0.01.

Western blotting analysis showed an increased expression of RIP-1 protein for both lineages after treatments with AcL3 and AcL4 (Figure 6A–D). All other evaluated proteins involved in apoptosis, Apaf-1, Bcl-2, caspase 7, and the key protein for autophagy, Beclin, did not show significant changes compared to the control. Furthermore, no bands were seen corresponding to cleaved PARP and cleaved caspase 8.

### 2.8. A. coriacea Fraction Increased TBARS Production, Inhibits Spheroids Growth and Induces Necroptosis by Overexpression of RIP-3 and AIF Proteins

We see that the levels of MDA were significantly (*p* < 0.05) increased (28%) after exposure to AcL3 fraction when compared to control (Figure 6E). Regarding tumoral growth, there was no significant difference in the size of the spheroids and in tumor cells spreading after AcL3 treatment in any observed times, while the control group increased by 34% in size after 24 h (*p* < 0.05) and 47% after 48 h (*p* < 0.005) of treatment (Figure 6F–H). Concerning proteins involved with the necroptotic pathway, we found increased expression of proteins RIP-3 and AIF proteins on U251 after treatment with AcL3 (Figure 6I,J).

## 3. Discussion

The Annonaceae family has a wide variety of chemical compounds [23,24], and acetogenins are secondary metabolites considered chemotaxonomic markers. Uvaricin [25] was the first cytotoxic isolated acetogenin and opened a new field of research. The *A. coriacea* fractions (AcL1-AcL4) were handled to obtain a higher acetogenin concentration, a process confirmed by the HPLC analysis (Table 1). Remarkably, the presence of acetogenins (Table 2 and Figure 4) validated the results found in this study, as metabolites of the classes of compounds (2c and 3a) found mainly in the most active fraction (AcL3) showed antitumor activity against several tumor cells [26]. By convention, for an extract to be promising as an antineoplastic, it must present an IC50 ≤ 30 µg/mL [27]. All *A. coriacea* fractions decreased cell viability showing IC50 ≤ 7 µg/mL, being AcL3 one of the most cytotoxic. This effect was confirmed by trypan blue and AO/PI assays (Figure 1).

In the present study, we also observed that AcL3 was able to prevent cell migration for both lineages (Figure 2) through decreased MMP-2 activity but not its expression (Figure 5). GBM cell migration has already been correlated with the action of some proteins, such as MMPs [28]. A study showed that an aqueous extract of *A. muricata* has already been described as presenting the acetogenin annoreticuin [29]. It is worth mentioning that the type of extraction used influences the total amount of acetogenins obtained as well as the results obtained. In addition, acetogenins have been described as molecules capable of strongly binding to calcium and magnesium ions, which is required for MMP activation [30]. Since *A. coriacea* fractions reduce the activity of MMP-2 but do not change its expression, and EDTA, a potent inhibitor of these endopeptidases, are also metal ion chelators [31], we can suggest a new possible mechanism of action for acetogenins, since there is no record associating the chelating capacity of acetogenins and MMPs inhibition.

We observed that all fractions were more selective for tumor cells than the chemotherapy drug TMZ. Selective index (SI) is an important parameter to determine the cytotoxic preference of a compound for tumoral cells compared to normal ones. TMZ has SI < 1 for both lineages, so we consider selective fractions with SI ≥ 1 (Figure 3). Thus, we further investigated cell death pathways modulated by fractions.

Of note, the RIP-1 protein was the only one with altered expression after exposure with the most selective fractions (Figure 6). In addition to the activation of RIP-1, we observed morphological alterations (Figure 1) characteristic of necroptosis, such as loss of membrane permeability and increase and extravasation of the cytoplasmic volume [32].

Necroptotic pathways are not fully elucidated yet, but there are some correlations between this type of death and ATP depletion [33]. Notably, acetogenins are considered potent inhibitors of complex I, thus leading to ATP depletion on the mitochondrial electron transport chain. This fact can justify the SI found since tumoral cells require more ATP compared to normal cells, so being more affected by its depletion [34]. However, the blockade of complex I by acetogenins is well described in the literature as being responsible for activating apoptosis [35]. Even so, there are few reports about the non-activation of apoptosis and/or activation of necroptosis, as found in this study. Previously, the extracts (C3) of *A. coriacea* that gave rise to AcL3 used in this work demonstrated that C3 induces cell death through DNA damage in human cervical cancer without triggering apoptosis [36]. In addition, the presence of acetogenins in *Annona muricata* leaf extracts has been reported as responsible for inducing pancreatic tumoral cells to necrosis [37]. Moreover, a synthetic mimetic of an acetogenin induced rectal adenocarcinoma cells through death (via caspase-independent pathway) due to the increase in intracellular ROS, complex I blockage, increase in RIP-1 and AIF translocation [38]. Therefore, we found that AcL3 increases the expression of RIP-3 and AIF, as well as the expression of MDA, a marker of oxidative stress (Figure 6). Thus, we suggest that acetogenins present in AcL3 can block complex I leading to ATP depletion and ROS increase, which may contribute to AIF translocation and triggering of necroptosis via necrosome formation (RIP-1/RIP-3).

Moreover, the evaluation of the action of *A. coriacea* fractions over the 3D extracellular microenvironment (spheroids) replicates the associated growth factors and signaling cascades found in vivo [39]. We observed that AcL3 was able to prevent tumor growth, and the cells spread over time (Figure 6). Once again, we can associate this fact with the inhibition of MMP-2, which may indicate that this fraction could prevent the invasive feature of GBM.

## 4. Materials and Methods

### 4.1. Plant Material and Extraction

The leaves of *A. coriacea* Mart (SISGEN: A11AE20) were collected in Catalão, GO, Brazil (18°09′16.4″ S; 47°55′43.2″ W) in May 2010 and identified by Prof. Hélder Nagai Consolaro from Federal University of Catalão. A voucher specimen (no 47919) was deposited at the Herbarium of Integrated laboratory of zoology, ecology, and botany at the Federal University of Goiás. The air-dried plant material (619 g) was pulverized in a knife mill, extracted at room temperature with ethanol (3 × 9 L, 7 days each), filtered, and concentrated to yield the crude leaves extract (CLE) (57.5 g, 9.2%). The liquid–liquid extraction was performed to fractionate the CLE using a 2 L separating funnel, with solubilization of the sample in 1 L of methanol/water (3:7, *v:v*) and extracted with hexane and after ethyl acetate (5 × 500 mL each) and the hexane (12.3 g), ethyl acetate (20.4 g), and hydroalcoholic (5.0 g) fractions were obtained, respectively.

The hexane fraction (12.3 g) was chromatographed on a silica gel (70–230 mesh) column (5.0 × 11.0 cm) with hexane/ethyl acetate/methanol gradient (1:0:0; 1:1:0; 0:1:0; 0:1:1; 0:0:1) to afforded five fractions (1–5). Fr. 4 (1.1 g) was separated into eight fractions (Fr. 4.1–4.8) on a Sephadex LH-20 column (2.5 × 40.0 cm) eluted with methanol (1.6 L) to provide the fraction 4.4 (0.6 mg) (AcL2).

The ethyl acetate fraction (20.4 g) was chromatographed on a silica gel (70–230 mesh) column (5.0 × 13.0 cm) with hexane/ethyl acetate/methanol gradient (1:0:0; 1:1:0; 0:1:0; 0:1:1; 0:0:1) to yield five fractions (6–10). Fr. 8 (8.2 g) was separated into five fractions (Fr. 8.1–8.5) on a silica gel (70−230 mesh) column (5.0 × 11.0 cm) eluted with hexane/ethyl acetate/methanol (1:0:0; 1:1:0; 0:1:0; 0:1:1; 0:0:1), and then Fr. 8.4 (1.5 g) was subjected to a Sephadex LH-20 column (2.5 × 40.0 cm) with methanol as eluent (1 L) to provide the fraction 8.4.3 (0.5 g) (AcL1). Fr. 9 (6.5 g) was separated into five fractions (Fr. 9.1–9.5) on a silica gel (70–230 mesh) column (4.0 × 12.0 cm) eluted with hexane/ethyl acetate/methanol (1:0:0; 1:1:0; 0:1:0; 0:1:1; 0:0:1). Fr. 9.4 (4.4 g) was subjected to a Sephadex LH-20 column (2.5 × 36.0 cm) with methanol (2 L) to provide the fraction 9.4.5 (0.8 g) (AcL3). Fr. 9.5 (1.2 g) was subjected to a Sephadex LH-20 column (2.5 × 45.0 cm) and eluted successively with methanol (1 L), providing the fraction 9.5.7 (0.3 g) (AcL4).

### 4.2. Chemical Characterization of the Acetogenins from A. coriacea Fractions

The chemical differentiation of the four fractions from A. coriacea (AcL1 to AcL4) was carried out by HPLC-ESI-HRMS/MS. Each sample (1.0 mg) was dissolved in methanol (1.0 mL) and filtered through a cellulose acetate filter (0.45 µm). The chromatographic separation was carried out using an NST 18 column (4.6 mm × 100 mm, 5.0 μm) at 20 °C with mobile phases of deionized water (A) and acetonitrile (B), both acidified with 0.1% formic acid. The applied gradient was 50 to 100% B over 55 min at a flow rate of 1.0 mL min-1 and a 10 µL injection volume. The MS parameters used were as follows: spray voltage of 4 kV, sheath gas flow rate of 30 arbitrary units, the auxiliary gas flow rate of 10 arbitrary units, capillary temperature of 350 °C, auxiliary gas heater temperature of 300 °C, S-lens 55, and collision energy offset of 20 eV. The samples were analyzed using the HR full-scan experiment set up in the *m*/*z* range of 150 to 750 Da to obtain MS/MS spectra [40]. The mass data were processed using MZmine 2, focusing on the intensity of the precursor ion (peak height) and the area of each peak, considering the baseline for deconvolution of 1.0 × 10^4^, 75% weight in *m*/*z* and 25% in RT for peak alignment.

### 4.3. Cell lines and Cultures and Treatments

The human GBM cell lines GAMG and U251 (ECACC, Salisbury, UK) and the normal human astrocyte cell line (NHA) (ATCC, LGC Promochem Middlesex, UK) were incubated in a humidified chamber with 5% CO_2_ at 37 °C and maintained in Dulbecco’s modified Eagle’s medium (DMEM; Sigma Aldrich, St. Louis, MO, USA), supplemented with 10% fetal bovine serum (FBS; Gibco-Life Technologies, Grand Island, NY, USA) and 1% penicillin/streptomycin. Cell line authentication was performed in June 2019, as previously reported [41]. The dissociation of confluent monolayers was made with 0.25% trypsin-EDTA (Life Technologies). IC_50_ values of the fractions were used in the assays.

### 4.4. MTT Assay and Selectivity Index

For in vitro testing, all the fractions were dissolved in dimethylsulfoxide (DMSO, Sigma, St. Louis, MO, USA) at a concentration of 25 µg/mL and stored in the dark at 4 °C.

The cells were seeded at a density of 5 × 10^3^ cells/well into 96-well plates and treated with increasing concentrations of the fractions (0, 1, 7, 15, and 25 µg/mL) in DMEM (0% FBS) and DMSO (1%) for 24 h. The vehicle DMSO (1%) was used as a control. Cell viability was quantified using the 3-(4,5-dimethylthiazol-2-yl)-2,5- diphenyltetrazolium bromide (MTT; Sigma, St. Louis, MO, USA) at 570 nm, and the IC_50_ was estimated by non-linear regression analysis. The selectivity index (SI) was calculated by the ratio of IC_50_ of the fractions in the normal cell line/IC_50_ of the same fraction against the tumoral cell line [42].

### 4.5. Trypan Blue Exclusion Viable Cell Assay

Membrane integrity and cell viability were also evaluated by trypan blue (0.04%) staining. After 24 h of treatment, the supernatant was discarded, and the staining solution was added. Then the cells were randomly photographed at 200× magnification on an inverted microscope (Axio Vert1 A1 FL—Carl Zeiss^®,^, Jena, Germany). The relative percentage of stained and non-stained cells of twenty pictures per well was analyzed and compared to the control.

### 4.6. Acridine Orange/Propidium Iodide Double Staining Cell Death Assessment

The cell death stages were analyzed and quantified by fluorescence assay using acridine orange (AO) and propidium iodide (PI) [43]. The results of the stages of cell death were transformed in percentage relative to the control.

### 4.7. Wound-Healing Assay

The wound-healing assay was performed using 24-well culture plates at a density of 5 × 10^5^ cells per well. After cells reached confluence, two scratches were made per well, using a sterile pipette tip (200 µL). The cells were washed twice with phosphate-buffered saline (PBS) before their subsequent incubation with treatments. Images were taken at 0, 24, 48, and 72 h after treatments on an inverted microscope and measured using ImageJ. The relative percentage of the reduction throughout time was analyzed and compared to the control. For morphological analysis, the images obtained in the bright field during wound-healing assay and in the dark field using a FITIC filter after AO/IP staining were used.

### 4.8. Chemical Characterization of the Acetogenins from A. coriacea Fractions

The chemical differentiation of the four fractions from *A. coriacea* (AcL1 to AcL4) was carried out by HPLC-ESI-HRMS/MS. Each sample (1.0 mg) was dissolved in methanol (1.0 mL) and filtered through a cellulose acetate filter (0.45 µm). The chromatographic separation was carried out using an NST 18 column (4.6 mm × 100 mm, 5.0 μm) at 20 °C with mobile phases of deionized water (A) and acetonitrile (B), both acidified with 0.1% formic acid. The applied gradient was 50 to 100% B over 55 min at a flow rate of 1.0 mL min^−1^ and a 10 µL injection volume. The MS parameters used were as follows: spray voltage of 4 kV, sheath gas flow rate of 30 arbitrary units, an auxiliary gas flow rate of 10 arbitrary units, the capillary temperature of 350 °C, auxiliary gas heater temperature of 300 °C, S-lens 55, and collision energy offset of 20 eV. The samples were analyzed using the HR full-scan experiment set up in the *m*/*z* range of 150 to 750 Da to obtain MS/MS spectra [40]. From the mass analysis of the fractions, AcL1 to AcL4 were afforded the total ion chromatograms with adequate separation of the acetogenins (Figure 5). The mass data were processed using MZmine 2, focusing on the intensity of the precursor ion (peak height) and the area of each peak, considering the baseline for deconvolution of 1.0E4, 75% weight in *m*/*z* and 25% in RT for peak alignment.

### 4.9. Zymography

We determine the action of *A. coriacea* fractions on MMPs cells supernatant (20 µL) after 24 h of treatment. In addition, we investigated the action of the fractions directly on the MMP-2 enzyme. For this, 3 µg/mL and 6 µg/mL of treatment (1% DMSO) were mixed with 2 µg/mL of MMP-2 in a non-denaturing sample buffer (125 mM SDS, 125 mM Tri-HCl, pH 6.8, 10% glycerol and 0.001% bromophenol blue). The Zymography was performed under non-reducing conditions through a 7% SDS-polyacrylamide gel containing 0.1% gelatin. After the running, gels were washed in 2.5% TritonX-100 for 60 min and then incubated at room temperature for 18 h in activation buffer (0.6% CaCl2, 0.05 M Tris-HCl, pH 8.0). Gels were stained for 1 h (0.25% Coomassie blue G-250, 30% ethanol, 10% acetic acid) and destained (30% ethanol, 10% acetic acid) for 2 h. The clear bands on a dark background, corresponding to the enzyme activity, were quantified on ImageJ and compared to the control [44].

### 4.10. Analysis of Protein Expression by Western Blotting

After the electrophoresis on SDS–PAGE, proteins were transferred to nitrocellulose membrane using semi-dry transfer TE 70 Semi-Dry Transfer Unit (Amersham ECL Semi-Dry Blotters—GE Healthcare Life Sciences, USA). The expression of MMP-2, Apaf-1, RIP-1, Bcl-2, Beclin, Caspase 7, cleaved PARP, and cleaved caspase 8, RIP-3, and AIF were evaluated. β-actin was used as a loading control reference protein. All the antibodies were diluted according to the manufacturer’s recommendations and were incubated, for 16 h, after the membrane blockage for 1 h with 5% skim milk powder. Subsequently, membranes were washed twice with TBS-T and incubated with the corresponding secondary antibody. Immunoreactive bands were visualized using chemiluminescence and analyzed using ImageJ. The relative expression of the target protein was normalized with the control band [45].

### 4.11. Expression of TBARS

The level of Thiobarbituric acid reactive substances (TBARs) was estimated by TBARs assay following the method with some adaptations [46]. U251 cells at a density of 22.5 × 10^5^ cells/well were cultivated in 75 cm^2^ flasks and treated with AcL3. Cell lysates were prepared by sonication, and the malondialdehyde (MDA) was quantified at 532 nm.

### 4.12. Spheroids Formation

We used the suspended drop method to generate the 3D culture [47]. U251 cells were seeded at a density of 3.0 × 10^5^ cells/mL at the upper surface of Petri dishes with PBS at the bottom to keep the moisture. After that, the dishes were kept immobile in incubators, at 37 °C and 5% CO_2_, for 72 h until the establishment of spheroids. Eight spheroids were transferred to a 12-well plate containing treatment diluted in DMEM (10% FBS). The spheroids were photographed every day for three days. The morphometric analysis was obtained by measuring the perimeter of the spheroid and the scattering of the cells using ImageJ.

### 4.13. Statistical Analysis

All results are expressed as mean ± standard deviation. One-way ANOVA followed by Tukey’s test was used for multiple comparisons. Paired t-tests were used to compare the means of two samples. All analyses were performed using GraphPad Prism version 7.0, and * *p* < 0.05, ** *p* < 0.01, *** *p* < 0.001, and **** *p* < 0.0001 were considered statistically significant.

## 5. Conclusions

Ethyl acetate fraction (AcL3) of *Annona coriacea* enriched with acetogenins are highly cytotoxic, prevent cell migration and MMP-2 activation, possibly due to their metal ion chelating properties, and may also induce necroptosis possibly by blocking complex I of the electron transport chain, increasing ROS production, leading to AIF translocation and necroptosome formation, triggering necroptosis on GBM cells. Despite our work correlating the presence of acetogenins in the fractions and the antitumor activity observed, for a more accurate statement, it would be necessary to carry out the experiments in tumor cells with these substances isolated. Moreover, further studies are warranted to interrogate the mechanisms of action triggered by acetogenins, especially those involving MMP inhibition and necroptosis activation.

## Figures and Tables

**Table 1:** Identification of acetogenins from *A. coriacea* fractions.

Retention Time	Compound	Fractions																			
			AcL3					AcL4					AcL1					AcL2			
			*m*/*z*	Erro (ppm)	Peak Height	Peak Area		*m*/*z*	Erro (ppm)	Peak Height	Peak Area		*m*/*z*	Erro (ppm)	Peak Height	Peak Area		*m*/*z*	Erro (ppm)	Peak Height	Peak Area
7.24	3a		645.45862	1.30	2.74 × 10^8^	2.16 × 10^9^		645.45795	0.26	3.94 × 10^8^	3.16 × 10^9^		645.45868	1.40	8.22 × 10^7^	1.13 × 10^9^		645.45831	0.82	9.1 × 10^7^	1.1 × 10^9^
15.96	5a		613.46798	0.05	1.2 × 10^8^	3.72 × 10^8^		613.46802	0.11	1.33 × 10^8^	2.05 × 10^8^		613.46875	1.31	7.51 × 10^7^	6.6 × 10^8^		-	-	-	-
17.24	5b		613.46887	1.50	4.99 × 10^7^	6.79 × 10^8^		613.46863	1.11	7.14 × 10^7^	9.58 × 10^8^		613.46838	0.71	3.10 × 10^7^	8.43 × 10^8^		-	-	-	-
18.95	5c		635.45056	1.05	2.81 × 10^7^	7.15 × 10^7^		635.44995	0.09	3.57 × 10^7^	8.09 × 10^8^		635.45050	0.96	1.94 × 10^7^	3.0 × 10^7^		-	-	-	-
22.70	1a		639.48425	1.01	7.20 × 10^7^	6.90 × 10^8^		639.48401	0.64	4.80 × 10^7^	5.20 × 10^8^		639.48407	0.74	7.40 × 10^7^	2.2 × 10^8^		639.48425	1.01	4.50 × 10^7^	1.80 × 10^9^
24.62	1c		661.46613	0.89	1.22 × 10^8^	1.88 × 10^8^		661.46552	−0.03	6.14 × 10^7^	1.15 × 10^9^		-	-	-	-		-	-	-	-
25.60	2a		619.45569	1.16	1.45 × 10^7^	4.92 × 10^8^		-	-	-	-		619.45593	1.54	4.13 × 10^7^	3.95 × 10^9^		619.45538	0.66	2.50 × 10^7^	4.00 × 10^8^
28.30	2b		597.47363	1.00	4.58 × 10^7^	1.41 × 10^8^		-	-	-	-		597.47351	0.80	3.03 × 10^8^	3.56 × 10^9^		-	-	-	-
29.17	2c		597.47406	1.73	1.10 × 10^8^	1.56 × 10^9^		597.47314	0.18	4.86 × 10^7^	9.45 × 10^8^		597.47406	1.72	2.24 × 10^8^	1.69 × 10^9^		597.47416	1.86	9.50 × 10^7^	2.80 × 10^8^

Green: compound detected; red: compound not detected.

**Table 2:** Metabolites of these classes of compounds have shown activity against various tumoral cells.

Classes of Compounds	Possible Metabolites	References
2c	annonacin, goniothalamicin. annonacin-A, annoreticuin, cis-annonacin, cis-goniothalamicin, arianacin, javoracin, glacin-A, glacin-B, asitrilobin-B, rolliacocin, asitrocin, montalicin-C, montalicin-D, cis-annoreticuim, montalicin-F	[10,11,12,13,14,15,16,17,18,19,20,21,22]
3a	coriheptocin-A, coriheptocin-B	-

## Data Availability

No new data were created or analyzed in this study. Data sharing is not applicable to this article.

## Sample Availability

Samples of the compounds are available from the corresponding author.
